# Does It Matter What Keeps You Awake? Effects of Two Different Sleep Deprivation Methods on Object‐Location Memory and Hippocampal c‐Fos Expression in Mice

**DOI:** 10.1111/jsr.70079

**Published:** 2025-04-23

**Authors:** Adithya Sarma, Mirthe Ronde, Soraya Smit, Peter Meerlo, Robbert Havekes

**Affiliations:** ^1^ Neurobiology Expertise Group Groningen Institute for Evolutionary Life Sciences (GELIFES), University of Groningen Groningen the Netherlands

**Keywords:** gentle handling, gentle stimulation, memory consolidation, novel objects, novelty, spatial memory

## Abstract

In sleep research, various sleep deprivation methods have been used to examine the effects of sleep loss on memory. However, studies often overlook the distinct impacts each method may have on activity in specific neuronal circuits and memory storage. It remains unclear whether these changes following sleep deprivation result from extended wakefulness alone or from an interaction with the nature of the waking experience. To address this question, we examined how two commonly used sleep deprivation methods in mice—gentle handling and novelty‐induced sleep deprivation—affect object‐location memory and hippocampal c‐Fos expression. Using either method, mice were sleep deprived for 3 or 6 h immediately after training in the object‐location memory task, and spatial memory performance was assessed 1 day after training. Object‐location memory was impaired after 3 and 6 h of novelty‐induced sleep deprivation, but only after 6 h of sleep deprivation by gentle handling. Assessing c‐Fos expression in separate groups of mice immediately after 3 or 6 h of sleep deprivation showed that both methods increased c‐Fos expression in the CA1 and CA3 regions after 3 h of sleep deprivation, while effects in the dentate gyrus depended on the method and blade examined. After 6 h of sleep deprivation, no significant changes in hippocampal c‐Fos expression were observed regardless of the method used. Overall, our findings show that the type of experience mice have while being kept awake and the duration of sleep deprivation can have different effects on spatial memory and neuronal activity in hippocampal subregions.

## 1 Introduction

Sleep is a phenomenon found across the phylogenetic tree that plays a crucial role in the overall survival of an individual (Gluckman et al. 2016; Joiner 2016; Keene and Duboue 2018). Consequently, a lack of sleep can have a significant impact on the health and well‐being of individuals. Numerous studies in human and animal models have demonstrated that sleep loss can impair key processes such as cognition, immune function and metabolism (Bryant et al. 2004; Irwin 2015; Kreutzmann et al. 2015; Spiegel et al. 2009). One important aspect of cognition that is affected by sleep deprivation (SD) is memory. Indeed, previous studies in rodent models have showcased that SD can significantly hamper hippocampus‐dependent memories. For example, it has been observed that even a brief period of total SD can attenuate fear memories (Delorme et al. 2019; Graves et al. 2003; Hagewoud, Whitcomb, et al. 2010; Hagewoud, Bultsma, et al. 2010; Vecsey et al. 2009) and working memories (Chauveau et al. 2014; Hagewoud, Havekes, et al. 2010). Additionally, several groups have observed that SD can also impair spatial memories such as object‐location memory (OLM) (Bolsius et al. 2023; Havekes et al. 2016; Raven, Heckman, et al. 2019).

The effects of SD on the hippocampus‐dependent memories in rodents mentioned above have been studied using a variety of different methods to keep the animals awake. One common method for inducing total SD that involves minimal sensory stimulation and disturbance is the so‐called gentle handling (GH) method, which is often based on gently tapping or shaking the cages when the rodents show signs of drowsiness (Borght et al. 2006; Hagewoud, Bultsma, et al. 2010). This SD method has been validated both behaviorally and through EEG recordings, demonstrating its effectiveness in keeping mice awake with minimal changes in cortisol levels (Meerlo and Turek 2001; Raven, Heckman, et al. 2019). Another common SD method relies on subjecting animals to novelty, which involves exposing them to new environments with or without objects to keep them awake, engaging their exploratory nature (Bellesi et al. 2013; Vyazovskiy et al. 2008). Like GH, this method has been validated in previous EEG studies, confirming its effectiveness in maintaining wakefulness (Bellesi et al. 2015, 2013). A key distinction between these two SD methodologies is that GH aims to minimise novelty and sensory stimulation, while novelty‐induced SD (NOV) depends on animals being aroused and active in the context of novel experiences.

It seems likely that different procedures of SD, in addition to keeping animals awake, may have method‐specific effects that depend on the sensory input involved and the brain circuitry that is activated. Indeed, SD methods such as NOV and GH have been shown to differentially impact brain regions such as the hippocampus. For example, NOV can enhance synaptic strength by increasing phosphorylation and protein levels of AMPA receptors, as well as axon‐spine interface and non‐perforated spines (de Vivo et al. 2017; Vyazovskiy et al. 2008). In contrast, GH can result in a net decrease in synaptic strength by impairing protein synthesis, causing spine loss, and impairing long‐lasting forms of long‐term potentiation in the hippocampus (Raven, Meerlo, et al. 2019; Tudor et al. 2016; Vecsey et al. 2009). Consequently, it is important to examine if GH and NOV impact hippocampus‐dependent memories differently, as this knowledge is essential for accurately interpreting the results of SD studies and their implications for memory research.

While the effects of GH on OLM are well‐studied, the effects of NOV on OLM are to our knowledge completely unknown. Although the methodologies are clearly specified in the literature, the collective body of research often generalises the effects of SD, without distinguishing the impacts that these different methods might have on memory. Therefore, we investigated the effects of GH vs. NOV on OLM and quantified c‐Fos expression in the hippocampus and determined whether the nature of the stimulus during SD can differentially affect memory consolidation and neuronal activity in the hippocampus.

## 2 Materials and Methods

### 2.1 Animals and Housing

Male C57Bl6/J mice (Charles River, Germany) were ordered at 6 weeks of age and pair‐housed on arrival in poly‐carb clear cages (Makrolon Type II‐L Cages) with stainless‐steel wired lids and sawdust as bedding material (Aspen). The animals were additionally provided with a paper roll and nesting material and had ad libitum access to food and water. Mice were individually housed 1 week before the start of the experiments when the animals were 9–12 weeks old. The experimental room was kept under a constant temperature (22°C ± 1°C), humidity (55% ± 10%) and a 12:12 h light/dark cycle. All procedures were approved by the national Central Authority for Scientific Procedures on Animals (CCD), the Institutional Animal Welfare Body (IvD, University of Groningen, the Netherlands) and conformed to Directive 2010/63/EU.

### 2.2 Experimental Groups and Design

In the first experiment, we assessed the impact of GH and NOV on OLM. Mice underwent OLM training at the start of the light phase. Immediately after training, mice were subjected to 3 or 6 h of SD using either GH or NOV, while the control groups were not sleep deprived (NSD). The mice then underwent a testing trial 24 h post‐acquisition (18 or 21 h post‐SD). In the second experiment, we explored the impact of GH and NOV on c‐Fos expression in the hippocampus. Mice were sleep deprived for 3 or 6 h at the start of the light phase using either GH or NOV. The NSD groups remained undisturbed. Brains were collected directly post‐SD for c‐Fos expression analysis.

### 2.3 Novel Object‐Location Memory Task

**FIGURE 1:**
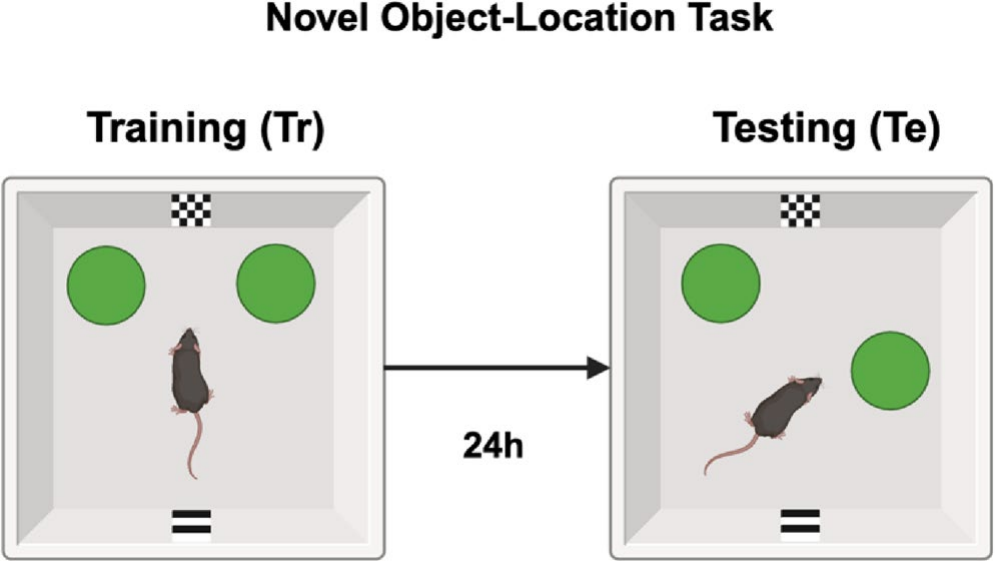
Schematic example of experimental set‐up in the novel object‐location paradigm. During training (Tr), mice can explore a spatial context with two identical objects located symmetrically to one another in space for 10 min. After a 24 h interval, mice enter a test phase (Te) which also lasts 10 min and where one object is displaced to a novel location.

The OLM is a hippocampus‐dependent spatial memory task that is based on the innate preference of a rodent for spatial novelty. In the present study, OLM was evaluated in a rectangular arena (length 40 cm, width of 30 cm and height of 30 cm) with two spatial cues at the short walls on opposite sides of the arena (Figure 1). The spatial cues covered the full short walls and consist of a black and white checkerboard pattern (total length × width: 25.4 cm × 21 cm, with black and white checkerboards of 1.5 cm × 1.5 cm) or alternating black and white striping (total length × width: 30 cm × 21 cm, with horizontal stripes that are 2.6 cm wide). The objects used were either two blue aluminium cylinders (height 12 cm and diameter 3.5 cm), two orange aluminium cylinders with tapering tops (height 12 cm and diameter 3.5 cm), two green glass cylinders (height 12 cm and diameter 2.5 cm), or two pink round vases (height 10 cm and diameter ranging from 3.5 cm at the bottom to 1.5 cm at the top). The objects were chosen such that they could not be moved by the animals.

The OLM paradigm consisted of two 10‐min trials taking place at the start of the light phase: an acquisition or training trial and a testing trial that took place 24 h later. Additionally, animals were habituated to the empty OLM arena for 5 min 1 day before the training trial. During the training trial, mice were allowed to freely explore two identical objects positioned symmetrically along a horizontal axis, each roughly 7.5 cm from the same wall of the arena (see Figure 1). Animals were placed into the arena on the same side, facing the wall and away from the objects. During the testing trial, one of the objects was displaced along a straight line to a position that was 15 cm away from the location of the object during the training trial.

The specific objects that the mice were exposed to were balanced within and between experimental groups to prevent confounding effects of object preference. Between animals and trials, the objects and arena were cleaned with a 70% ethanol solution to avoid the presence of olfactory cues. The exploration times per mouse for each object during the training and test trial were manually scored using BORIS (BORIS 7.12.2; (Friard and Gamba 2016)) by two experimenters blind to treatment. Directing the nose to the object at a distance of no more than 1 cm and/or touching the object with the nose was considered exploratory behaviour (Bolsius et al. 2023; Heckman et al. 2020). These exploration times were used to calculate if the mice discriminated between the displaced and non‐displaced object during the test session, that is, the discrimination index (d2): (relocated object—non‐relocated object)/(relocated object + non‐relocated object). The d2 index is a relative measure of discrimination corrected for total exploration time and can range from −1 to 1. A significant difference from zero, that is, chance level, indicates that the mice remembered the object locations from the training session, and a difference from the control condition signifies an actual effect on memory performance by SD. Animals were excluded from the experiment if they climbed out of the arena during testing (1 from the NSD group and 1 from the NOV group in the 3‐h SD experiment).

### 2.4 Sleep Deprivation Procedures

Mice were subjected to a single SD period of 3 or 6 h directly following the acquisition trial of the OLM, or at the start of the light phase for immunohistochemical experiments. SD was performed via the GH or NOV. Mice that were sleep deprived using the GH method were kept awake by gently tapping or shaking the cages when they showed signs of drowsiness (Hagewoud, Bultsma, et al. 2010). This method has been validated at the behavioural level and through EEG recordings (Meerlo et al. 2001), and it has been substantiated by several laboratories that the observed behavioural and plasticity phenotypes associated with SD were not caused by elevated plasma corticosterone levels or by the GH itself (Raven, Heckman, et al. 2019). Mice sleep deprived via the introduction of novelty were transferred to a novel cage and presented with a novel object when they presented signs of drowsiness (Bellesi et al. 2013; Vyazovskiy et al. 2008). Novel objects included small rubber balls, wooden blocks, plastic, metallic and wooden objects in different shapes and colours. To prevent potential interference between the OLM task and the NOV sleep deprivation procedure, the objects used during the OLM task were not reused during sleep deprivation and were distinct both in terms of shape and physical context. Objects were cleaned with ethanol between sessions to avoid the presence of olfactory stimuli. This method was validated by previous EEG studies as well (Bellesi et al. 2015, 2013). Prior to the experiments, the experimenters handled all the mice in the SD room for 4 min per day for at least three consecutive days.

### 2.5 Perfusions and Tissue Collection

For immunohistochemical analyses, animals were sacrificed immediately post‐SD. Mice were perfused transcardially under 100 μL of pentobarbital to minimise discomfort. The mouse was first flushed with around 150 mL of 0.9% NaCl‐Heparin solution and then fixated with 150 mL of 4% paraformaldehyde. Brains were removed and post‐fixated in 10 mL cups filled with 4% PFA for 24 h at room temperature. The brains were then washed with 0.01 M PB, after which they were immersed in 30% sucrose for cryopreservation. When the brains sunk to the bottom of the cup, they were flash‐frozen using liquid nitrogen and stored at −80°C. 20 μm frozen hippocampal slices were then cut in the coronal plane (ranging from bregma coordinate −1.2 to approximately −3.4) using a cryostat (Leica CM3050 or Leica CM 1860, Leica Biosystems, Wetzlar, Germany) and were stored free‐floating in cups containing PBS with 1% sodium azide at 4°C.

### 2.6 Immunohistochemistry

Immunohistochemical DAB (3,3′‐diaminobenzidine) stainings were performed on hippocampal tissue sections to visualise c‐Fos expression. Cups containing 8 (4 dorsal and 4 ventral) sections each were washed 3× with 0.01 M PBS for 5 min and then blocked with 0.3% H_2_O_2_ in PBS for 10 min. After washing, sections were pre‐incubated with 0.3% Triton X‐100 and 5% normal goat serum for 1 h. Then, sections were incubated with the primary antibody anti‐c‐Fos (1:1000, Abcam ab208942, LOT: GR3437625‐1) in PBS containing 5% normal goat serum and 0.3% Triton X‐100 for 3 nights of incubation at 4°C. After washing away the primary antibody solution, the sections were incubated with a secondary antibody (1:400, Biotinylated goat‐anti‐ mouse IgG; LOT: 147137) in PBS for 2 h. Subsequently, the sections were extensively washed and incubated with the avidin‐biotin (AB) complex (1:500) in PBS for 2 h (Vectastain PK‐6100 standard, Vector Laboratories, USA). Then, the sections were washed with PBS before continuing with the DAB step. DAB solution was prepared by dissolving DAB tablets in Milli‐Q. The reaction was activated by adding 0.1% H_2_O_2_ to each sample and stopped by a series of washes with the buffer. The sections were mounted on Superfrost microscope slides (LOT: 1189, Thermo Scientific) with the help of 1% gelatine after which they were left to dry for a minimum of 24 h. After drying, the sections mounted on glass slides were dehydrated through graded ethanol and then cleared in Xylol before being cover slipped with DPX mounting medium. The sections were visualised using an optical light microscope (Olympus CX23LEDRFS1, Olympus Corporation, Japan), and the numbers of c‐Fos‐positive cells were counted manually.

### 2.7 Statistical Analyses

Statistical analyses and design of graphs were done using Graphpad Prism 10 (GraphPad Software, La Jolla, CA). The data obtained were checked for normality using a Shapiro–Wilk test and checked for equal variances by using Bartlett's test. If the data for discrimination index and total exploration times were found to be normally distributed, the groups were compared and analysed using a one‐way ANOVA. A post hoc analysis was conducted in the form of a Tukey's honestly significant difference (HSD) test when the ANOVA showcased significance. For data that did not have a normal distribution, a Kruskal–Wallis test was used. If the Kruskal–Wallis test showed a significant outcome, post hoc analysis was conducted by using Dunn's multiple comparison test. Differences between exploration times of relocated and non‐relocated objects were carried out using a two‐way ANOVA with repeated measures and a post hoc comparison was performed using the Bonferroni's multiple comparison. One sample *t*‐tests were performed to assess whether the d2 index for each condition differed significantly from zero (chance level) in the OLM. Results were considered statistically significant when *p* < 0.05. The data were presented in bar plots ± SEM.

## 3 Results

### 3.1 Object‐Location Memory Impairment Is Dependent on Duration and Method of Sleep Deprivation

**FIGURE 2:**
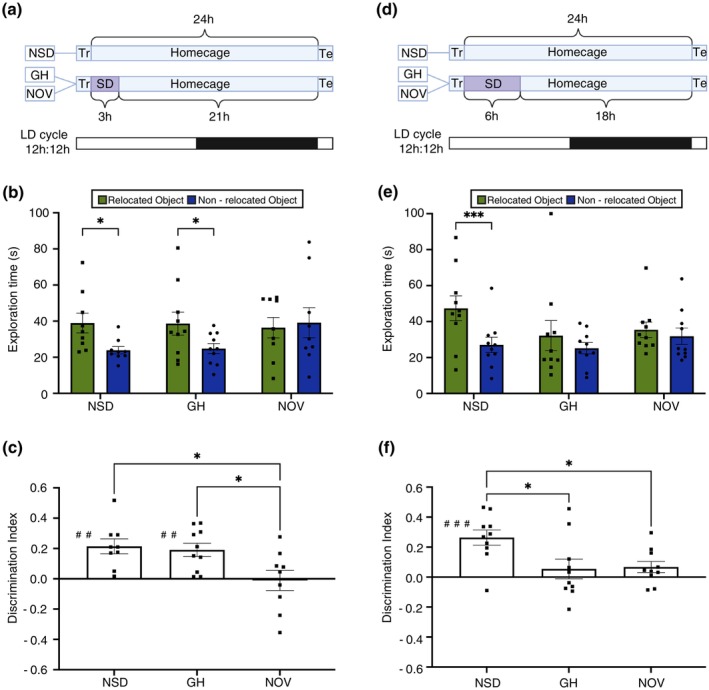
Effects of sleep deprivation duration and method on spatial memory performance in the OLM task. (A) Mice were subjected to novelty induced‐sleep deprivation (NOV) or gentle handling (GH) for 3 h immediately after training (Tr) at the start of the light phase, while a control group was allowed to sleep (NSD). Spatial memory was assessed 24 h later during the test (Te) session. (B) NOV significantly impaired spatial memory, as indicated by the absence of increased exploration of the relocated object (*p < 0.05, two‐way ANOVA with repeated measures; *N* = 9–10). (C) The impairment in the NOV group is further demonstrated by the discrimination index, which shows no significant preference for either object, while both the GH and NSD groups exhibited a significant preference for the relocated object (# indicates a significant difference from zero, with a positive index reflecting a preference for the relocated object and a negative index reflecting a preference for the non‐relocated object; ##*p* < 0.01, independent sample *t*‐test. * indicates a significant difference between groups, *p < 0.05, one‐way ANOVA; *N* = 9–10). Data is presented as bar plots with mean ± SEM. (D) A separate group of mice were subjected to GH or NOV for 6 h immediately after training at the start of the light phase, while the NSD groups were left undisturbed. Spatial memory was assessed 24 h later during the testing session. (E) 6 h of GH or NOV significantly impaired spatial memory, as indicated by the absence of increased exploration of the relocated object (***p < 0.001, two‐way ANOVA with repeated measures; *N* = 9–10). (F) The impairment is further demonstrated by the discrimination index, with both GH and NOV groups showing no significant preference for the relocated object, while the NSD group exhibited a significant preference. (# indicates a significant difference from zero, ###*p* < 0.001, independent sample *t*‐test; * indicates a significant difference between groups, *p < 0.05, one‐way ANOVA; *N* = 9–10). Data are presented as bar plots with mean ± SEM.

Previous research has demonstrated that as little as 3 h of NOV can significantly alter neural plasticity in brain regions critical for long‐term spatial memory (Vyazovskiy et al. 2008; Xie et al. 2016). However, the direct impact of this brief period of SD on long‐term spatial memory has not been explored, nor has it been compared with the effects of GH on long‐term spatial memory. Here, we aimed to address these gaps by comparing the effects of 3 h of NOV and GH on long‐term spatial memory, using the OLM task. We subjected the mice to 3 h of SD using GH or NOV at the beginning of the light phase directly following the training session. The memory was assessed 24 h after training (Figure 2A). Our observations indicate that mice performed significantly worse when they had been sleep deprived by novelty in comparison to NSD and GH groups, evidenced by differences in exploration times of the relocated object (exploration time of relocated vs. non‐relocated object, two‐way ANOVA with repeated measures, *p < 0.05, Figure 2B) and differences in d2 index (one‐way ANOVA, *p < 0.05). Furthermore, we found that the d2 index of NSD and GH groups significantly differed from zero, indicating successful recognition of the relocated object (## represents a significant difference from zero, one sample *t*‐test, *p* < 0.01, Figure 2C). In contrast, the d2 index of NOV group did not differ from zero, suggesting an inability to remember that an object has been moved (N.S., Figure 2C). Moreover, the effects of SD on memory were not attributed to total exploration times during testing (T1 NSD = 76.98 ± 9.09 vs. T1 GH = 82.08 ± 9.32 vs. T1 NOV = 82.56 ± 7.03; *p* = 0.88, N.S.; T2 NSD = 62.91 ± 6.74 vs. T2 GH = 63.52 ± 8.80 vs. T2 NOV = 75.61 ± 12.47; *p* = 0.59, N.S.). These findings reveal that as little as 3 h of NOV but not GH can significantly impair OLM, demonstrating that the impact of SD on hippocampus‐dependent spatial memory varies per method.

Numerous studies, including ours, have focused on the effects of 6 h SD using GH on hippocampus‐dependent memories such as OLM (Bolsius et al. 2023; Heckman et al. 2020). However, there has been no direct comparison of how GH and NOV differentially affect memory when deprived for 6 h. This comparison is particularly important, considering our observed differences in their impact following 3 h of deprivation. Therefore, we subjected mice to 6 h of SD using GH or NOV at the beginning of the light phase directly following T1 (Figure 2D). We found that mice deprived of sleep for 6 h by GH or NOV failed to discriminate the relocated object from the non‐relocated object during the test session (### represents a significant difference from zero, one sample *t*‐test, *p* < 0.001; Figure 2F). Additional statistical analyses confirmed that mice performed significantly worse under SD conditions (exploration time of relocated vs. non‐relocated object, two‐way ANOVA with repeated measures, ***p < 0.001, Figure 2E, d2 index, one‐way ANOVA, *p < 0.05, Figure 2F). Moreover, the observed impairments were not a consequence of alterations in total exploration times (T1 NSD = 63.1 ± 10.9 vs. T1 GH = 65.05 ± 12.58 vs. T1 NOV = 74.24 ± 8.16; *p* = 0.34, N.S.; T2 NSD = 74.4 ± 10.7 vs. T2 GH = 57.4 ± 10.9 vs. T2 NOV = 67.2 ± 8.5; *p* = 0.50, N.S.).

Our results demonstrate that the effects of SD on hippocampus‐dependent spatial memory vary with the method and duration of deprivation. Notably, while GH impairs memory performance only after 6 h, even 3 h of SD through novelty exposure is sufficient to disrupt OLM. This pattern highlights the differential impacts of SD method and duration on spatial memory in mice.

### 3.2 c‐Fos Expression in the Hippocampus Is Dependent on Duration and Method of Sleep Deprivation

**FIGURE 3:**
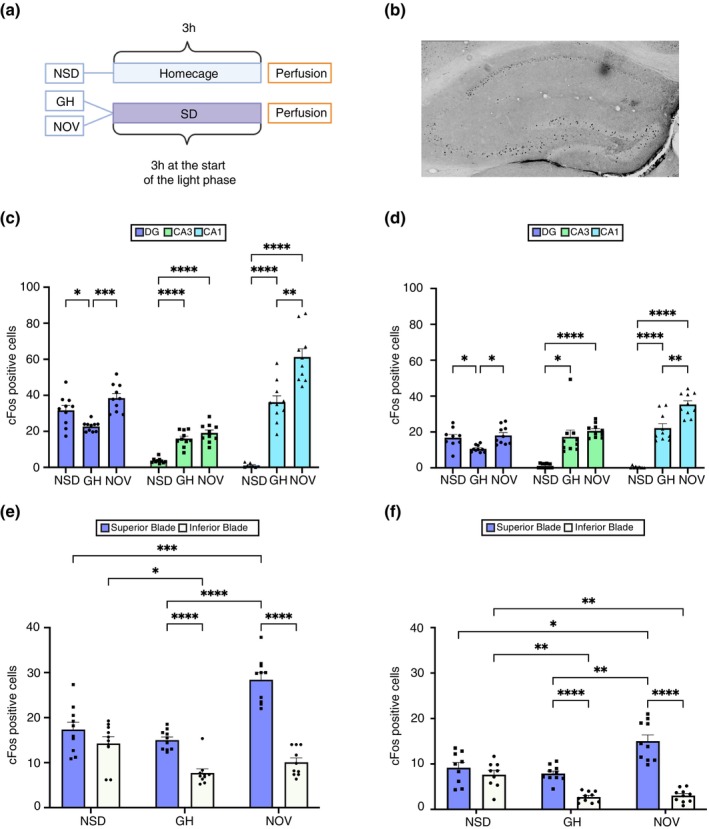
3 h of sleep deprivation by gentle handling and novelty exposure differentially affect c‐Fos expression in the dentate gyrus. (A) Mice were subjected to 3 h of sleep deprivation via gentle handling (GH) or novelty (NOV) at the start of the light phase, while a control group was allowed to sleep (NSD). Mice were perfused immediately after the 3‐h sleep deprivation period. c‐Fos expression was assessed in the dentate gyrus (DG), CA3, and CA1 regions. (B) Representative image to showcase the immunohistochemical DAB staining for c‐Fos after 3 h of SD (scale 50 μm, Magnification 20×). (C) In the dorsal hippocampus (dHC), c‐Fos expression significantly differed between conditions (mixed effects model, *p* < 0.001). GH decreased c‐Fos expression in the DG compared to NSD (*p < 0.05) and NOV (****p < 0.0001), while NOV did not alter DG c‐Fos levels compared to NSD (*p* > 0.05, n.s.). In the CA3 and CA1 regions, both GH and NOV significantly increased c‐Fos expression compared to NSD (****p < 0.0001), with NOV showing higher c‐Fos expression than GH in CA1 (**p < 0.01). (D) In the ventral hippocampus (vHC), significant differences were found across conditions (mixed effects model, *p* < 0.001). GH reduced c‐Fos expression in the DG compared to NSD (*p < 0.05), while NOV significantly increased c‐Fos levels compared to GH (****p < 0.0001). In CA3 and CA1 regions, both GH and NOV significantly increased c‐Fos expression compared to NSD (****p < 0.0001), with NOV showing higher c‐Fos levels than GH in CA1 (**p < 0.01). (E) In the dorsal DG blades, c‐Fos expression was significantly higher in the superior blade compared to the inferior blade for all SD groups (two‐way ANOVA, ****p < 0.0001). NOV increased c‐Fos expression in the superior blade compared to NSD (**p < 0.01), and showed a trend towards decreased expression in the inferior blade (*p* = 0.06). GH decreased c‐Fos in the inferior blade compared to NSD (*p < 0.05). (F) In the ventral DG blades, c‐Fos expression in the superior blade was significantly higher than in the inferior blade across all sleep deprived groups (two‐way ANOVA, ****p < 0.0001). NOV increased c‐Fos expression in the superior blade compared to NSD (**p < 0.01) and decreased it in the inferior blade (**p < 0.01). GH selectively decreased c‐Fos expression in the inferior blade compared to NSD (**p < 0.01).

To determine if 3 h of SD induced by GH or NOV differentially affect the hippocampus, we analysed c‐Fos expression across the dorsal (dHC) and ventral hippocampus (vHC) (Figure 3A). Our findings highlight significant differences in how each SD method modulates c‐Fos expression across the dentate gyrus (DG), CA3, and CA1 regions (Figure 3B–D). In the DG, GH significantly decreased c‐Fos expression in comparison to the NSD group in both the dorsal and ventral hippocampus (*p* < 0.05, Figure 3C,D). In contrast, NOV left c‐Fos expression in the DG unaltered compared to NSD and was associated with significantly higher c‐Fos levels compared to GH (*p* < 0.0001, Figure 3C,D). Conversely, in the CA3 and CA1 regions, both SD methods significantly increased c‐Fos expression in comparison to NSD (dHC, CA3: *p* < 0.0001, CA1: *p* < 0.0001; vHC, NSD vs. NOV: *p* < 0.0001, NSD vs. GH: *p* < 0.05, Figure 3C,D). Furthermore, c‐Fos expression in CA1 post‐NOV was significantly higher than after GH induced SD (*p* < 0.01, Figure 3C,D). Altogether, these results demonstrate that the method of SD can significantly influence the extent of c‐Fos expression in the hippocampus. While both methods significantly increased c‐Fos expression in the CA3 and CA1 regions, GH reduced total c‐Fos levels in the DG, whereas NOV did not alter total c‐Fos levels in the DG.

To investigate the effect of sleep loss on the DG in detail, we examined whether a particular SD method can have a more profound effect on either of the two blades of the DG. Our observations revealed that in the dorsal and ventral DG, both SD methods were associated with increased c‐Fos expression in the superior blade in comparison to the inferior blade (*p* < 0.0001, Figure 3E,F). Notably, in the dorsal DG, NOV increased c‐Fos expression in the superior blade in comparison to the NSD group (*p* < 0.01, Figure 3E) while it showed a trend toward a decrease (*p* = 0.06, Figure 3E) in the inferior blade. On the other hand, GH only decreased c‐Fos expression in the inferior blade in comparison to the NSD group (*p* < 0.05, Figure 3E). Similarly, in the ventral DG, NOV increased c‐Fos expression in the superior blade in comparison to the NSD group (*p* < 0.01, Figure 3F) and decreased c‐Fos expression in the inferior blade (*p* < 0.05, Figure 3F), while GH only decreased c‐Fos expression in the inferior blade in comparison to the NSD group (*p* < 0.01, Figure 3F). These findings indicate that while GH specifically suppressed c‐Fos expression in the inferior blade, NOV enhanced expression in the superior blade and reduced it in the inferior blade, underscoring the differential impacts of SD methods on hippocampal c‐Fos expression.

**FIGURE 4:**
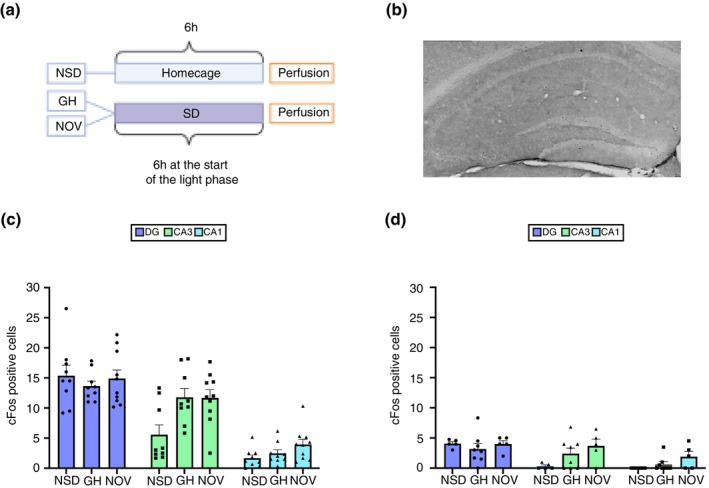
6 h of sleep deprivation, irrespective of the method used, does not alter c‐Fos expression in the hippocampus. (A) Mice were subjected to 6 h of novelty‐induced sleep deprivation (NOV) or gentle handling (GH) at the start of the light phase, while a control group was allowed to sleep (NSD). c‐Fos expression was assessed in the dentate gyrus (DG), CA3, and CA1 regions. (B) Representative image to showcase the immunohistochemical DAB staining for c‐Fos after 6 h of SD (Scale 50um, Magnification 20×). (C) In the dorsal hippocampus (dHC), no significant differences in c‐Fos expression were found in the DG between the NSD, GH, and NOV groups (Tukey's multiple comparisons, *p* > 0.05). In CA3, there was a trend towards higher c‐Fos expression in the sleep deprived groups compared to NSD, but this did not reach significance (NSD vs. NOV: *p* = 0.0661; NSD vs. GH: *p* = 0.0565). No significant differences were observed in CA1 between groups (*p* > 0.05). (D) In the ventral hippocampus (vHC), c‐Fos expression did not significantly differ between NSD, GH, and NOV in the DG (*p* > 0.05). In CA3, there was a trend towards higher c‐Fos expression in the NOV group compared to NSD (*p* = 0.0700), but no significant differences were found between the other groups. No significant differences were found in CA1 between the groups (*p* > 0.05).

To examine whether 6 h of SD induced by GH or NOV affects hippocampal activity, we analysed c‐Fos expression across the dHC and vHC (Figure 4A). In the DG, neither GH nor NOV significantly altered c‐Fos expression compared to the NSD group (dHC, NSD vs. GH: *p* = 0.65, NSD vs. NOV: *p* = 0.99, Figure 4C; vHC, NSD vs. GH, *p* = 0.99; NSD vs. NOV, *p* = 0.99, Figure 4D). Similarly, in the CA3 and CA1 regions, c‐Fos expression remained unaltered by both SD methods. In the CA3 region, c‐Fos expression showed a trend toward an increase in both the GH and NOV compared to NSD, but these changes did not reach statistical significance (dHC: NSD vs. GH, *p* = 0.06, NSD vs. NOV: *p* = 0.07, Figure 4C; vHC: NSD vs. GH, *p* = 0.19, NSD vs. NOV: *p* = 0.07, Figure 4D). In the CA1 region, c‐Fos expression was also unaffected by either method of SD (dHC: NSD vs. GH, *p* = 0.11; NSD vs. NOV, *p* = 0.16, Figure 4C; vHC: NSD vs. GH, *p* = 0.45; NSD vs. NOV, *p* = 0.19, Figure 4D). Altogether, the results indicate that 6 h of SD did not alter c‐Fos expression in the hippocampus marking a clear distinction between the effects of 3 and 6 h of SD on hippocampal c‐Fos expression. While 3 h of SD led to method‐specific alterations in c‐Fos expression across the hippocampus, no significant changes were observed after 6 h of SD, regardless of the method used. This suggests that, unlike short‐term SD, prolonged SD may not differentially impact neuronal activation as measured by c‐Fos expression, as levels remain consistent with those of the NSD group.

## 4 Discussion

It is a widely accepted notion that sleep is essential, whereas SD is detrimental to memory. However, close examination of the literature suggests that the impact of sleep loss on brain regions important for memory, such as the hippocampus, can vary depending on the methods and durations used to keep animals awake (Havekes and Aton 2020; Sarma and Havekes 2024). For example, NOV involves heightened arousal and can enhance synaptic strength, while GH is designed to minimise sensory stimulation and novelty and is associated with decreased synaptic strength (Raven, Heckman, et al. 2019; Vyazovskiy et al. 2008). These fundamental differences underscore our long‐standing hypothesis that the inconsistencies reported across the literature may reflect the specific method and duration of SD used (Havekes and Aton 2020). Therefore, our primary goal here was to systematically compare GH and NOV side by side, assessing whether these two commonly used SD protocols produce divergent effects on hippocampus‐dependent OLM and hippocampal c‐Fos expression in mice.

We observed that SD for 3 h by NOV impaired OLM, whereas GH for the same duration did not. This observation that 3 h of NOV impaired memory while 3 h of GH did not may seem surprising given reports that shorter durations of GH have been linked to memory impairments. However, these different outcomes are likely due to key variations in the experimental design of these studies. For example, some studies found memory deficits when they trained animals, sleep deprived them for 2 h using GH and tested them directly thereafter (Binder et al. 2012; Sawangjit et al. 2018). This design captures acute effects of sleep loss such as altered motivation or exploratory drive rather than isolating effects on memory consolidation and retrieval. In contrast, by introducing a recovery period of 18 h before testing, our study focuses specifically on the effects of SD on memory consolidation, minimising potential confounds related to immediate performance. Interestingly, Sawangjit et al. reported memory impairments in OLM even after 2 h of SD by GH when the animals were tested 1 week later. These results, which at first glance might appear conflicting with our lack of deficits observed following 3 h of SD by GH, are difficult to compare directly due to the differences in time points tested (i.e., 24 h vs. 7 days). However, the two results in combination suggest that brief SD periods of 2–3 h may weaken memory traces in a way that only becomes apparent at longer delays. In other words, the absence of deficits at 24 h in our study may reflect a memory that is still sufficiently intact at that time point, but may be more vulnerable over longer intervals. In line with our study, Prince et al. (2014) reported that a 3‐h period of GH immediately post‐training did not impair memory when tested 24 h later. Altogether, the work by Prince et al. and our current study further underscores the importance of considering the duration of the sleep deprivation period in sleep studies. Extending the duration of SD to 6 h resulted in impaired OLM, irrespective of the SD method that was used. It is important to note that these differences in how different SD methods affect memory processes at 3 and 6 h cannot be attributed to circadian effects. This is because OLM training and testing were consistently conducted at the start of the light phase, with SD occurring immediately after training. A plausible explanation for the effects we observed after 3 h of SD on OLM may lie in the distinct nature of the SD methods used. As highlighted in previous studies, novelty exposure engages active exploration and sensory processing, which could lead to increased plasticity in several brain regions including the hippocampus (Bernstein et al. 2019; Staiger et al. 2000; VanElzakker et al. 2008). This heightened activity in addition to the effects of sleep loss may interfere with the memory consolidation processes needed to consolidate hippocampus‐dependent memories, such as OLM. In contrast, the GH method involves minimal sensory stimulation, which may allow animals to remain awake over shorter durations of SD without overwhelming hippocampal circuits or significantly interfering with memory consolidation processes. After 6 h of SD, the memory impairments observed with both methods likely reflect the cumulative effects of prolonged wakefulness, which can disrupt the normal functioning of hippocampal circuits over time. This supports the view that extended periods of total SD disrupt hippocampus‐dependent memory (Havekes and Aton 2020). Altogether, this pattern highlights that the effects of SD on memory depend on the combination of the SD method used and the duration of deprivation. Specifically, after 3 h of SD, NOV significantly impaired OLM, while GH did not. However, after 6 h of SD, both methods resulted in impaired memory. This indicates that both the type of experience during SD and the length of that experience contribute to the resulting memory deficits, and the relative contribution of each factor may shift depending on the method employed. Future memory studies could incorporate EEG recordings to measure sleep architecture, particularly rebound sleep after 3 and 6 h of SD, to assess how the buildup of sleep pressure, and perhaps local changes herein, may contribute to these impairments.

At the cellular level, immunohistochemical analysis revealed that 3 h of SD, regardless of method, increased c‐Fos expression in the CA1 and CA3 regions of the hippocampus. This aligns with the view that SD for brief periods can increase neuronal activity and plasticity in these areas, which are critical for hippocampal function (Vyazovskiy et al. 2008). In contrast to CA1 and CA3, the DG showed method‐specific responses, aligning with the principle that different brain regions often do not respond uniformly to the effects of SD (Havekes et al. 2016; Havekes and Aton 2020; Sarma and Havekes 2024; Vecsey et al. 2009). Specifically, c‐Fos levels in the DG were similar in NSD and NOV groups while they were significantly lower in the GH group. Furthermore, NOV increased c‐Fos expression in the superior blade of the DG, while GH specifically decreased c‐Fos expression in the inferior blade. This distinction may suggest that novelty itself could be driving the increased c‐Fos levels in the superior blade, as novelty is known to induce synaptic potentiation and c‐Fos expression in the DG, a region particularly responsive to new stimuli (Bernstein et al. 2019; Kitchigina et al. 1997). In contrast, the minimally stimulating GH method may lead to a more subtle engagement of novelty‐sensitive circuits, only leading to reduced neuronal activation caused predominantly by sleep loss. Therefore, the observed differences between the two methods in the DG could reflect the added effect of novelty exposure. In conclusion, it is important to recognise that SD may not affect different sub‐regions of the hippocampus in a similar manner, as evidenced by its effects on CA1 and CA3, where both methods increased c‐Fos, versus the divergent effects on the DG. Moreover, different SD methods can have distinct effects on specific regions, as illustrated by the opposing responses to GH and NOV in the DG.

Previous literature using undisturbed animals indicated that hippocampal c‐Fos levels exhibit relatively lower levels at ZT6 compared to ZT0 or ZT3 (Pantazopoulos et al. 2011). We observed that non‐sleep deprived mice showed lower c‐Fos levels in the dentate gyrus (Figure S1A,B), but no differences in CA3 and CA1 (Figure S1C–F) when comparing ZT6 to ZT3. When we summate the c‐Fos expression of the three hippocampal sub‐regions together and compare their levels between the two time points, we see a decrease in hippocampal c‐Fos expression. This likely reflects the modulation of c‐Fos expression by circadian rhythms, aligning with previous findings from Pantazopoulos et al. (2011). In addition, our data also indicate that different types of SD can alter natural fluctuations of c‐Fos in the hippocampus in a sub‐region‐specific manner. For instance, in the DG, GH for 3 h lowered c‐Fos compared to NSD, whereas NOV did not—a distinction that disappeared by the 6‐h mark (Figure S1A,B). In the dorsal CA3 region, we observed that after 3 and 6 h of SD, irrespective of the method used, c‐Fos expression remained higher than NSD at their respective time points (Figure S1C). Conversely, in CA1, the robust increase in c‐Fos seen after 3 h of SD by both methods was not sustained at 6 h (Figure S1E,F). These findings underscore that the nature (GH vs. NOV) and duration (3 vs. 6 h) of SD can produce differential c‐Fos responses across hippocampal sub‐regions.

Although circadian factors likely contribute to the overall lower c‐Fos at ZT6 in NSD animals, the regional differences in the SD groups suggest that method‐specific and duration‐dependent mechanisms are also at play, at least at the level of c‐Fos in the hippocampus. These findings are consistent with the scenario proposed by Havekes and Aton (2020), in which different SD methodologies—each entailing varying degrees of novel sensory input—can lead to varying effects in the brain. This conceptual framework aligns well with the sub‐region‐specific c‐Fos patterns we observe here and may help explain why different protocols result in divergent outcomes for memory and neuronal activity. As c‐Fos levels naturally fluctuate across the circadian cycle in undisturbed animals reaching their lowest at ZT6 and peaking at ZT18 (Pantazopoulos et al. 2011), future studies incorporating multiple time points may provide further insight into the interplay between SD effects and circadian rhythms.

Future studies should also consider measuring c‐Fos expression in mice subjected to SD following a learning task, as this may better capture how neuronal activity is modulated by prior experience. Nevertheless, the purpose of this study was to primarily examine how different SD methods affect OLM and the hippocampus, aligning with our work and previous studies on the effects of SD on plasticity (Diering et al. 2017; Havekes et al. 2016; Heckman et al. 2020; Raven, Meerlo, et al. 2019; Vecsey et al. 2009; de Vivo et al. 2017; Vyazovskiy et al. 2008). While the hippocampus may be a critical region for SD‐induced memory deficits, it is also important to recognise that c‐Fos expression is not restricted to the hippocampus. Therefore, it might be worth examining other brain regions involved in OLM, such as the medial prefrontal cortex and entorhinal cortex, which could provide a more comprehensive understanding of how different SD methods affect brain plasticity (VanElzakker et al. 2008; Zimmermann and Eschen 2017). More broadly, our current work lays the foundation for future studies investigating the impact of different SD methods on brain‐wide Fos expression patterns. Such investigations would allow researchers to test the hypothesis that distinct sleep deprivation methods lead to unique plasticity changes in a region‐specific manner, thereby potentially helping to resolve some of the discrepancies reported in the literature. In summary, the duration and method of SD are crucial factors in determining the effects on OLM and neuronal activity in the hippocampus. Our findings emphasise the need to consider both factors when interpreting the outcomes of SD studies and their broader implications for memory research.

## Author Contributions

**Adithya Sarma:** conceptualization, investigation, writing – original draft, writing – review and editing, formal analysis, visualization, methodology, data curation. **Mirthe Ronde:** investigation, writing – review and editing, formal analysis. **Soraya Smit:** investigation, formal analysis. **Peter Meerlo:** conceptualization, writing – review and editing, supervision. **Robbert Havekes:** conceptualization, writing – review and editing, funding acquisition, project administration, supervision.

## Conflicts of Interest

The authors declare no conflicts of interest.

## Supporting information


**Figure S1.** Comparative expression of c‐Fos in hippocampal sub‐regions across 3 and 6 h of sleep deprivation. (A) In the dorsal dentate gyrus (DG), c‐Fos expression significantly differed between conditions and timepoints (two‐way ANOVA, *p* < 0.001). In all three groups of mice, c‐Fos expression was significantly lower after 6 h compared to 3 h (NSD: *p* < 0.0001; GH: *p* < 0.01; NOV: *p* < 0.0001). Within the 3‐h timepoint, GH mice showed lower c‐Fos expression compared to NSD (*p* < 0.01), while NOV group did not differ significantly from NSD mice (*p* = 0.06, n.s.). At the 6‐h timepoint, c‐Fos expression did not differ significantly between groups (*p* > 0.05, n.s.). (B) In the ventral DG, c‐Fos expression also significantly differed between conditions and timepoints (two‐way ANOVA, *p* < 0.001). In all three groups, c‐Fos expression was significantly lower after 6 h compared to 3 h (NSD: *p* < 0.0001; GH: *p* < 0.001; NOV: *p* < 0.0001). Within the 3‐h timepoint, GH mice showed lower c‐Fos expression compared to NSD (*p* < 0.01), while NOV mice did not differ significantly from NSD mice (*p* > 0.05, n.s.). At the 6‐h timepoint, c‐Fos expression did not differ significantly between groups (*p* > 0.05, n.s.). (C) In the dorsal CA3, c‐Fos expression significantly differed between conditions and timepoints (two‐way ANOVA, *p* < 0.001). In the NOV group, c‐Fos expression was significantly lower after 6 h compared to 3 h (*p* < 0.001), while c‐Fos levels in NSD and GH groups did not significantly differ between the two timepoints (*p* > 0.05, n.s.). Within the 3‐h timepoint, both GH and NOV mice showed significantly higher c‐Fos expression compared to NSD (*p* < 0.0001 for both). At the 6‐h timepoint, both GH and NOV mice still showed significantly higher c‐Fos expression compared to NSD (*p* < 0.05 for both), with no significant differences between GH and NOV at either timepoint (*p* > 0.05, n.s.). (D) In the ventral CA3, c‐Fos expression significantly differed between conditions and timepoints (two‐way ANOVA, *p* < 0.001). In both GH and NOV groups, c‐Fos expression was significantly lower after 6 h compared to 3 h (GH: *p* < 0.0001; NOV: *p* < 0.0001), while c‐Fos expression in NSD mice did not significantly differ between timepoints (*p* > 0.05, n.s.). Within the 3‐h timepoint, both GH and NOV mice showed significantly higher c‐Fos expression compared to NSD (*p* < 0.0001 for both), while GH and NOV did not differ significantly from each other (*p* > 0.05, n.s.). At the 6‐h timepoint, c‐Fos expression did not significantly differ between groups (*p* > 0.05, n.s.). (E) In the dorsal CA1, c‐Fos expression significantly differed between conditions and timepoints (two‐way ANOVA, *p* < 0.0001). In both GH and NOV groups, c‐Fos expression was significantly lower after 6 h compared to 3 h (GH: *p* < 0.0001; NOV: *p* < 0.0001), while c‐Fos expression in NSD mice did not significantly differ between timepoints (*p* > 0.05, n.s.). Within the 3‐h timepoint, both GH and NOV mice showed significantly higher c‐Fos expression compared to NSD (*p* < 0.0001 for both), with NOV mice also showing significantly higher c‐Fos expression compared to GH mice (*p* < 0.0001). At the 6‐h timepoint, c‐Fos expression did not differ significantly between groups (*p* > 0.05, n.s.). (F) In the ventral CA1, c‐Fos expression significantly differed between conditions and timepoints (two‐way ANOVA, *p* < 0.0001). In both GH and NOV groups, c‐Fos expression was significantly lower after 6 h compared to 3 h (GH: *p* < 0.0001; NOV: *p* < 0.0001), while c‐Fos expression in NSD mice did not significantly differ between timepoints (*p* > 0.05, n.s.). Within the 3‐h timepoint, both GH and NOV mice showed significantly higher c‐Fos expression compared to NSD (*p* < 0.0001 for both), with NOV mice also showing significantly higher c‐Fos expression compared to GH mice (*p* < 0.0001). At the 6‐h timepoint, c‐Fos expression did not differ significantly between groups (*p* > 0.05, n.s.).

**Figure S2.** (A) Dorsal Hippocampus: Representative images of c‐Fos immunohistochemical DAB staining in the dorsal DG, CA3, and CA1 of the hippocampus for the NSD group and the GH and NOV groups after 3 h of sleep deprivation (scale: 50 μm, magnification: 20×). (B) Ventral Hippocampus: Representative images of c‐Fos immunohistochemical DAB staining in the ventral DG, CA3, and CA1 of the hippocampus for the NSD group and the GH and NOV groups after 3 h of sleep deprivation (scale: 50 μm, magnification: 20×).

**Figure S3.** (A) Dorsal Hippocampus: Representative images of c‐Fos immunohistochemical DAB staining in the dorsal DG, CA3, and CA1 of the hippocampus for the NSD group and the GH and NOV groups after 6 h of sleep deprivation (scale: 50 μm, magnification: 20×). (B) Ventral Hippocampus: Representative images of c‐Fos immunohistochemical DAB staining in the ventral DG, CA3, and CA1 of the hippocampus for the NSD group and the GH and NOV groups after 3 h of sleep deprivation (scale: 50 μm, magnification: 20×).

## Data Availability

The data that support the findings of this study are available from the corresponding author upon reasonable request.
