# Supplementary figures and images for: Does It Matter What Keeps You Awake? Effects of Two Different Sleep Deprivation Methods on Object‐Location Memory and Hippocampal c‐Fos Expression in Mice
Source: J Sleep Res. 2025 Apr 23;35(1):e70079. doi: 10.1111/jsr.70079 (PMC12856129; doi:10.1111/jsr.70079)

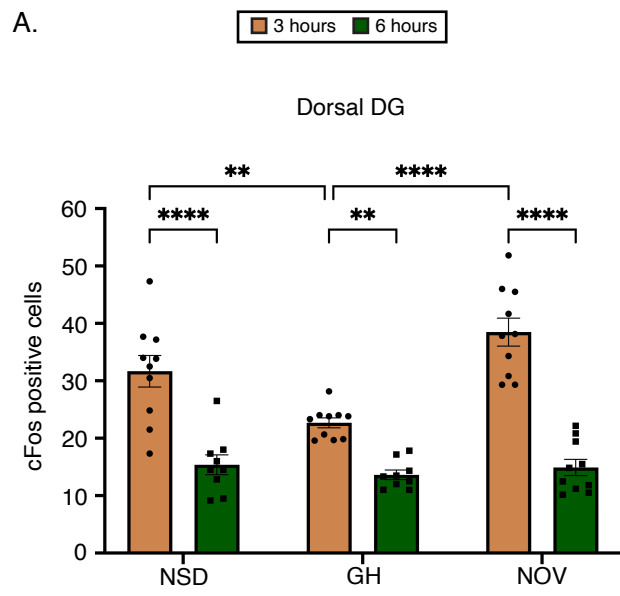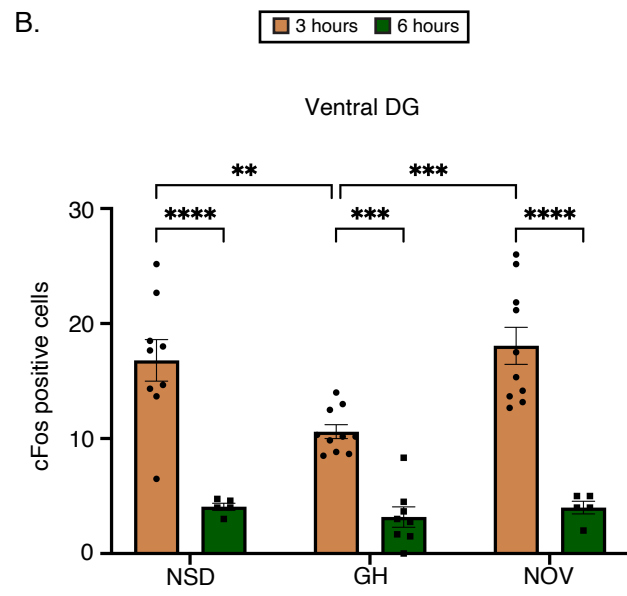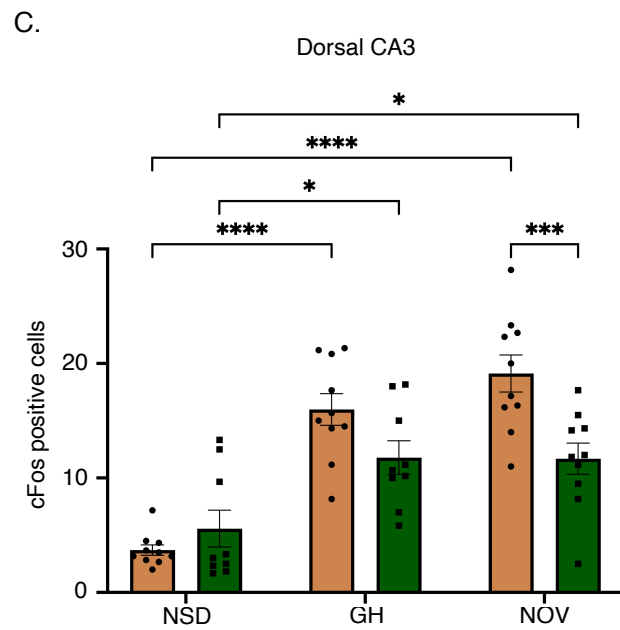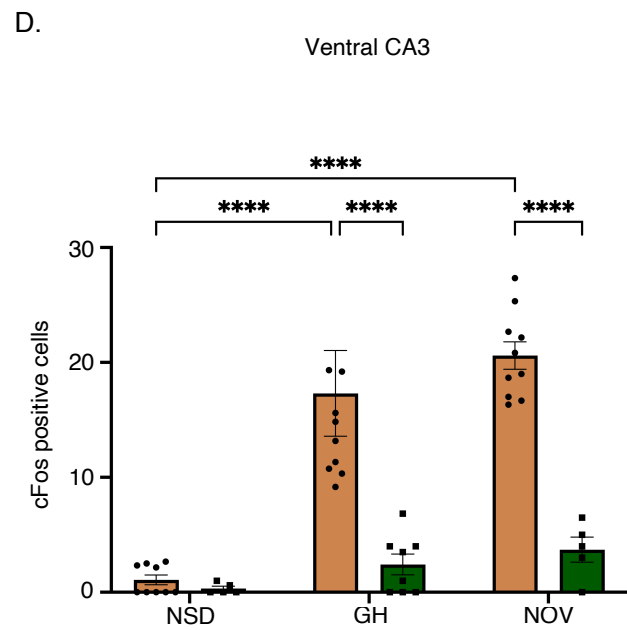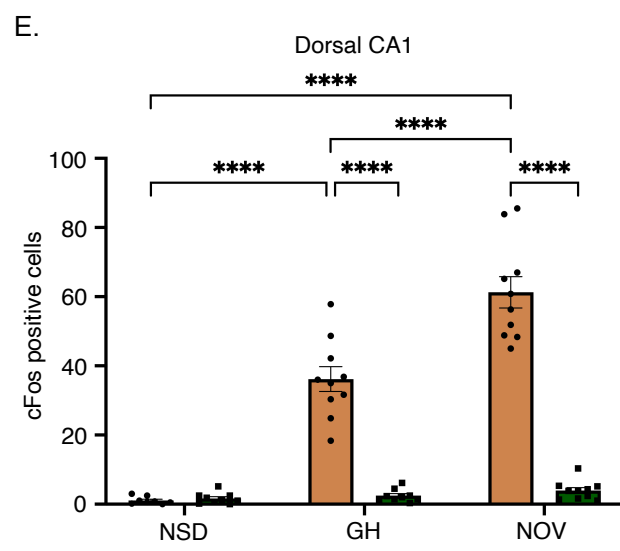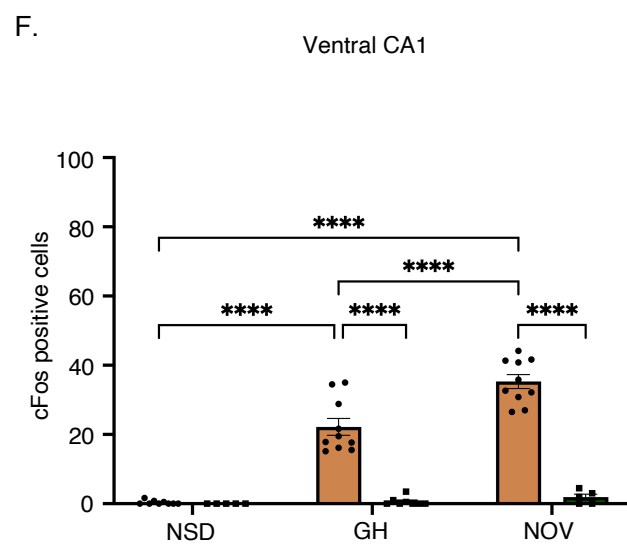

Supplement: Supplementary file 1 — Figure S1. Comparative expression of c‐Fos in hippocampal sub‐regions across 3 and 6 h of sleep deprivation. (A) In the dorsal dentate gyrus (DG), c‐Fos expression significantly differed between conditions and timepoints (two‐way ANOVA, p < 0.001). In all three groups of mice, c‐Fos expression was significantly lower after 6 h compared to 3 h (NSD: p < 0.0001; GH: p < 0.01; NOV: p < 0.0001). Within the 3‐h timepoint, GH mice showed lower c‐Fos expression compared to NSD (p < 0.01), while NOV group did not differ significantly from NSD mice (p = 0.06, n.s.). At the 6‐h timepoint, c‐Fos expression did not differ significantly between groups (p > 0.05, n.s.). (B) In the ventral DG, c‐Fos expression also significantly differed between conditions and timepoints (two‐way ANOVA, p < 0.001). In all three groups, c‐Fos expression was significantly lower after 6 h compared to 3 h (NSD: p < 0.0001; GH: p < 0.001; NOV: p < 0.0001). Within the 3‐h timepoint, GH mice showed lower c‐Fos expression compared to NSD (p < 0.01), while NOV mice did not differ significantly from NSD mice (p > 0.05, n.s.). At the 6‐h timepoint, c‐Fos expression did not differ significantly between groups (p > 0.05, n.s.). (C) In the dorsal CA3, c‐Fos expression significantly differed between conditions and timepoints (two‐way ANOVA, p < 0.001). In the NOV group, c‐Fos expression was significantly lower after 6 h compared to 3 h (p < 0.001), while c‐Fos levels in NSD and GH groups did not significantly differ between the two timepoints (p > 0.05, n.s.). Within the 3‐h timepoint, both GH and NOV mice showed significantly higher c‐Fos expression compared to NSD (p < 0.0001 for both). At the 6‐h timepoint, both GH and NOV mice still showed significantly higher c‐Fos expression compared to NSD (p < 0.05 for both), with no significant differences between GH and NOV at either timepoint (p > 0.05, n.s.). (D) In the ventral CA3, c‐Fos expression significantly differed between conditions and timepoints (two‐way AN [file JSR-35-e70079-s003.pdf]
